# A Simple High-Throughput Field Sample Preparation Method Based on Matrix-Induced Sugaring-Out for the Simultaneous Determination of 5-Hydroxymethylfurfural and Phenolic Compounds in Honey

**DOI:** 10.3390/molecules27238373

**Published:** 2022-11-30

**Authors:** Xijuan Tu, Fengjie Yu, Qian Jin, Chunping Du, Jiaxu Chen, Ji Yang, Yuchang He, Shaokang Huang, Wenbin Chen

**Affiliations:** 1 College of Animal Science (College of Bee Science), Fujian Agriculture and Forestry University, Fuzhou 350002, China; 2 MOE Engineering Research Center of Bee Products Processing and Application, Fujian Agriculture and Forestry University, Fuzhou 350002, China; 3 College of Food Science, Fujian Agriculture and Forestry University, Fuzhou 350002, China

**Keywords:** field sample preparation, high-throughput, homogenous liquid-liquid extraction, matrix-induced sugaring-out, honey

## Abstract

In the present work, a high-throughput field sample preparation method was reported for the simultaneous determination of 5-hydroxymethylfurfural and phenolic compounds in honey. Combining a simple and green homogenous liquid–liquid extraction, matrix-induced sugaring-out, with the use of a 96-deepwell plate and multichannel pipette, the proposed method showed its merits in instrument-free and high-throughput preparation. Due to the high-throughput property, the parameters of the method were rapidly and systematically studied using a constructed 4 × 2 × 4 × 3 array (sample amount × ratio of ACN:H_2_O × standing time × replicates) in a 96-deepwell plate. Analytical performance was fully validated, and the limits of detection and limits of quantification were in the range of 0.17–1.35 μg/g and 0.51–4.14 μg/g, respectively. Recoveries were between 83.98 and 117.11%, and all the precisions were <5%. Furthermore, the developed method was successfully applied in the outdoor preparation of commercial honey samples and the in-field preparation of raw honey samples in apiary. The current work presented a simple, rapid, and high-throughput method for the field sample preparation of honey and provides a valuable strategy for the design of field and on-site sample preparation.

## 1. Introduction

Honey, the natural sweet food produced by honeybees, has been long consumed by humans due to its special flavor and nutritional benefits [1]. Fructose and glucose are the major components of honey, with total content >60% (*w/w*) [2]. However, honey is more than a simple concentrated sweet substance given its diverse constituents from both plant and honeybee. These constituents including flavor compounds, organic acids, vitamins, proteins and amino acids, phenolic compounds, lipids, minerals, and other specific phytochemicals of botanic origin [3,4,5,6,7,8]. Since the honey matrix is complex, a sample preparation procedure prior to analysis is often required to reduce interferences and enrich analytes for this challenging analytical sample [9].

Homogenous liquid–liquid extraction (HLLE) is a widely used sample preparation technique based on the traditional liquid–liquid extraction (LLE) [10,11]. In HLLE, water miscible organic solvent is mixed with water to form a homogenous solution for extracting analytes. Subsequently, phase-separation agents or conditions are introduced to trigger the partition of organic solvent from its aqueous solution. After phase separation, multiple analytes with a wide range of polarities can be rapidly and efficiently extracted into the separated organic phase [12,13,14,15]. Moreover, the phase separation also makes high-polarity matrix compounds e.g., proteins [16] and sugars [17] mostly stay at the aqueous phase, which significantly reduces the interference. In addition, the obtained extractive solution is compatible with dispersive solid-phase extraction (d-SPE) [18,19], disposable pipette extraction (DPX) [20], matrix solid-phase dispersion [21], and dispersive liquid–liquid micro-extraction (DLLME) [22,23,24,25] for further clean-up or enrichment. Furthermore, the emerging solvents ionic liquids and deep eutectic solvent have been successfully used in HLLE and extended the applications of HLLE in micro-extraction [26,27].

Recently, we developed a novel HLLE method named matrix-induced sugaring-out (MISO) for the determination of neonicotinoid pesticides in honey [28]. The proposed MISO method takes advantage of constituents in sample matrix, the high contents of sugars in honey, to induce the phase separation of acetonitrile–water homogenous solution. Therefore, the general step of introducing a phase-separation agent in conventional HLLE is eliminated, which makes MISO a simple, fast, and low-cost HLLE method. This idea of matrix-induced HLLE has found application in another food matrix [29]. In the present work, MISO is further developed into a high-throughput field HLLE method for the simultaneous determination of HMF and phenolic compounds, which are the indicator of freshness [30,31] and the important functional constituents [6] in honey, respectively. The proposed method provides a simple and instrument-free strategy for the rapid field preparation of honey sample.

## 2. Results and Discussion

### 2.1. High-Throughput Field Matrix-Induced Sugaring-Out

**Figure 1:**
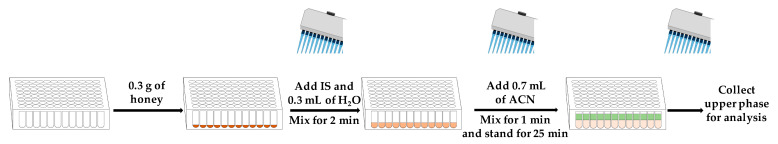
Schematic presentation of high-throughput field matrix-induced sugaring-out.

The strategy of high-throughput field matrix-induced sugaring-out (HT-F-MISO) is shown in Figure 1. The 96-deepwell plate and multichannel pipette were selected to make the sample preparation very simple and suitable for high-throughput field operation. In the HT-F-MISO, honey samples were weighed in the 96-deepwell plate with a portable balance, then water and acetonitrile (ACN) were added and mixed sequentially. After standing, the mixture was separated into two phases due to the MISO. The multiple analytes were extracted into the upper ACN phase, and the field sample preparation was completed after collecting the ACN phase for HPLC analysis.

The present method provides the advantages of simple and rapid preparation and the low consumption of samples and solvents. After optimization, 96 samples can be prepared within 60 min in one well-plate. Thus, the average consumption time for each sample is as low as only 40 s. In addition, the preparation method also exhibits the characteristics of miniaturization. The amount of sample is 0.3 g, which effectively reduces the generation of waste, and the preparation of one sample only requires 0.7 mL of ACN, which also effectively reduces the consumption of organic solvents in the protocol.

### 2.2. High-Throughput Optimization of Method Parameters

Since the high-throughput property, parameters of the HT-F-MISO was rapidly optimized using the sample array in a 96-deepwell plate.

Honey sample was mixed with H_2_O and ACN sequentially using multichannel pipette. In the original MISO method [28], ACN-H_2_O mixture was added to the honey sample, then homogenized by vortex device was carried out, whereas in field preparation, the use of electrical instruments should be avoided; thus, the multichannel pipette was used to perform homogenization in the HT-F-MISO. Because of the high viscosity, honey sample was mixed with H_2_O and ACN sequentially rather than mixed with ACN-H_2_O mixture for better homogenization. After adding water, honey sample could be well homogenized in 2 min. Then ACN was added and mixed with the aqueous honey solution with 1 min for completing homogenization.

Our previous study indicated that a high concentration of ACN in an ACN-H_2_O mixture was critical to obtain high extraction yields for polar compounds [13]. Therefore, ACN and H_2_O with volume ratios of 6:4 and 7:3 were investigated in the array optimization. Under these two concentrations, the amount of honey sample plays a crucial role in the phase separation: when sample mass was larger than 0.2 g, phase separation could be clearly observed. However, as the sample mass increased to greater than 0.6 g, insoluble substances in the lower phase emerged. Thus, the amount of sample was studied in the range between 0.2–0.5 g with increment of 0.1 g.

Additionally, standing for a certain time after homogenization was necessary for achieving stable phase separation. Clear two-phase solution could be observed after standing for 5 min, and thus, the effects of standing time on the recovery were investigated at 5, 15, 25, and 35 min.

**Figure 2:**
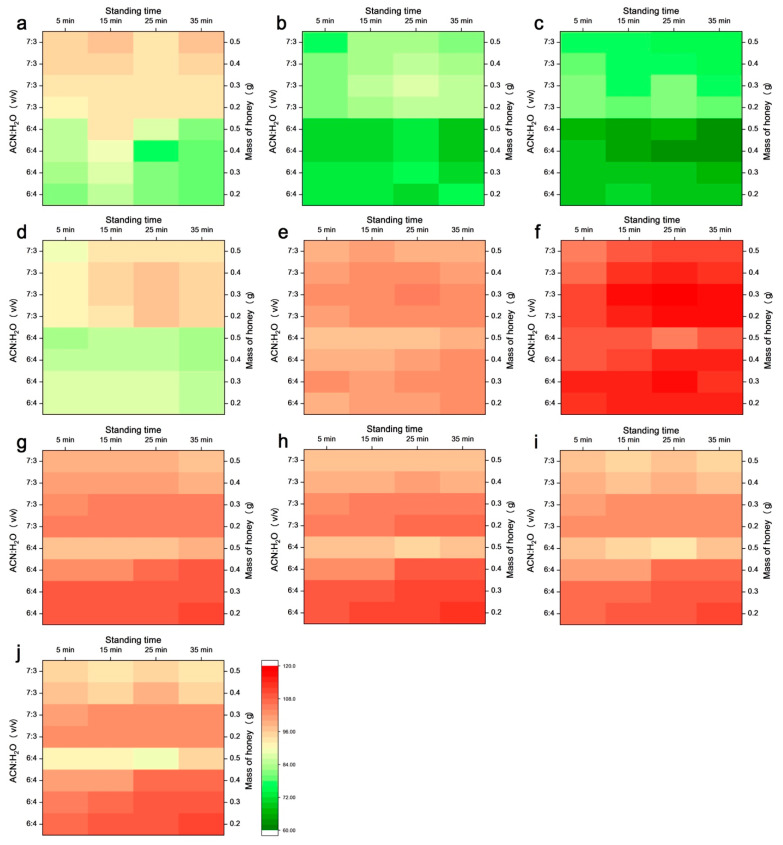
Effects of the ratio of ACN-H_2_O (6:4 and 7:3, *v*/*v*), mass of honey sample (0.2, 0.3, 0.4, and 0.5 g), and standing time (5, 15, 25, and 35 min) on the calculated recovery (%) of analytes. (**a**) 5-hydroxymethylfurfural; (**b**) protocatechuic acid; (**c**) pinocembrin; (**d**) caffeic acid; (**e**) 3,4-dimethoxycinnamic acid; (**f**) quercetin; (**g**) apigenin; (**h**) kaempferol; (**i**) chrysin; (**j**) galangin.

Based on the above considerations, we constructed a 4 × 2 × 4 × 3 array (sample amount × ratio of ACN:H_2_O × standing time × replicates), with a total of 96 samples in a 96-deepwell plate to perform the high-throughput optimization of the preparation parameters. Calculated recoveries of the HMF and the nine phenolic compounds under different conditions are summarized in Figure 2.

It can be seen from Figure 2 that when the ratio of ACN:H_2_O is 7:3 (*v*/*v*), recoveries of the analytes with lower retention time (Figure 2a–e) are higher than that at the ratio of 6:4 (*v*/*v*). This result is consistent with our previous research finding that increasing the concentration of ACN in ACN-H_2_O mixture results in the improvement of extraction yield, especially for compounds with high polarity [13]. For less-polar compounds (Figure 2f–j), high recovery was achieved at both 7:3 and 6:4 (*v*/*v*).

Additionally, prolonging the standing time is beneficial to the improvement of recovery. Results indicated that recoveries increased and reached the plateau in 25 min, which indicated that stable phase separation of HT-F-MISO could be achieved in 25 min without the assist of centrifugation instrument.

The sample amount of honey plays an important role in the HT-F-MISO. As shown in Figure 2, recoveries of most analytes reach the highest value at the mass of 0.3 g. With further increases in sample amount, the recoveries reduced slightly. This may be due to the low water content in the ACN-H_2_O mixture, which leads to the limitation of dissolvability for honey sample. This speculation could be supported by the observation that with further increasing the sample mass to be larger than 0.6 g, insoluble solid matter in the lower phase appeared after standing. Therefore, 0.3 g of honey sample was suitable for the HT-F-MISO to obtain a stable and high recovery.

In short, based on the above results obtained from the constructed array, the optimal protocol could be determined as 0.3 g of honey sample mixed with 0.3 mL of H_2_O for 2 min followed by mixing with 0.7 mL of ACN for another 1 min, then standing for 25 min for phase separation.

### 2.3. Analytical Performance

**Figure 3:**
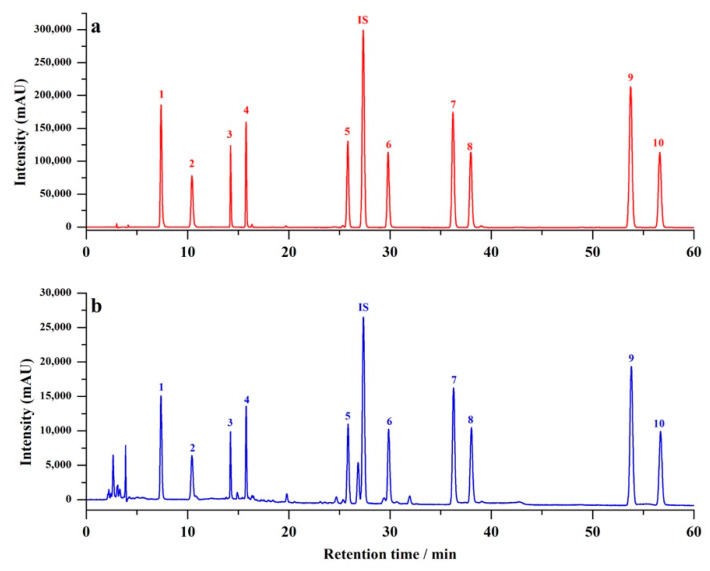
Representative chromatogram of (**a**) standards and (**b**) extractive solution from spiked honey sample. Peak 1, 5-hydroxymethylfurfural; 2, protocatechuic acid; 3, pinocembrin; 4, caffeic acid; 5, 3,4-dimethoxycinnamic acid; 6, quercetin; 7, apigenin; 8, kaempferol; 9, chrysin; 10, galangin; internal standard (IS), coumarin.

Reversed-phase HPLC separation was modified based on the literature [32]. Coumarin was used as the internal standard for quantification, which had proved to be suitable for the simultaneous measurement of multiple phenolic compounds in HLLE [33]. It can be seen from Figure 3 that all ten target analytes and the internal standard are well separated. The linearity range and the linear equation of calibration curves are shown in Table 1. Good linearity was obtained with coefficients (r^2^) all larger than 0.9989. The limits of detection (LODs, S/N = 3) and limits of quantification (LOQs, S/N = 10) in honey sample were in the ranges of 0.17–1.35 μg/g and 0.51–4.14 μg/g, respectively. The accuracy and precision were evaluated by recovery using a 3 × 6 array (3 spiked levels × 6 replicates) in a 96-deepwell plate. As summarized in Table 2, the obtained recoveries in intra-day and inter-day experiments were between 83.98 and 117.11%. Additionally, the precisions were all <5%. These results indicated that this simple, rapid, and high-throughput field sample preparation method could provide satisfactory analytical performance.

### 2.4. Real Samples Analysis

The validated HT-F-MISO method was applied in commercial and raw honey samples (Table 3). Twelve commercial samples (C1-C12) were purchased from market and prepared using a 12 × 3 array (samples × replicates) in outdoor environment to simulate field condition. Raw honey samples (R1-R6) were collected in six honeybee colonies in an apiary. The collected raw honey samples were in-field prepared in the apiary using a 6 × 3 array (samples × replicates). The obtained extractive solutions after preparing outdoor and in-field were brought to laboratory for HPLC analysis. Results shown that the detected contents of HMF were ranged from 1.85 μg/g to 85.88 μg/g in the commercial samples, in which three samples were found over the regulated level (40 μg/g) [2]. In contrast, the detected contents of HMF in the raw samples were all below LOQ. It is valuable to point out that the formation of HMF comes from the thermal dehydration of sugars [34], and the concentration of HMF in honey increases gradually along with the processing and storage [35,36,37]. Results obtained from the real samples indicated that HMF in the fresh honey was at very low level, whereas in commercial samples, heat treatment and long-term storage could lead to significant increases in HMF concentration. More importantly, since the phase separation of HLLE dramatically decreases the residual content of sugars in the extractive [17], this field sample preparation may reduce the impact of the sample transport process on the promotion of HMF production. In addition, four phenolic compounds were also detected in the samples, including protocatechuic acid, caffeic acid, and kaempferol in three commercial samples and 3,4-dimethoxycinnamic acid in four raw samples.

## 3. Materials and Methods

### 3.1. Chemicals and Materials

HPLC grade acetonitrile (ACN) and methanol were obtained from Merck (Darmstadt, Germany). Formic acid was purchased from Sinopharm Chemical Reagent Co., Ltd. (Shanghai, China). Standards of 5-hydroxymethylfurfural (HMF), protocatechuic acid, pinocembrin, caffeic acid, 3,4-dimethoxycinnamic acid, quercetin, apigenin, kaempferol, chrysin, galangin, and coumarin (internal standard, IS) were from Aladdin (Shanghai, China). Ultrapure water (18.2 MΩ·cm) was used through this article. Honey sample used in the method development was collected in Putian, China. Commercial honey samples in the method application were purchased from local market. The raw honey samples for field preparation were collected and performed in an apiary located in Jiufeng Moutain, Fuzhou, China. Stock solution of standards were prepared in methanol with concentration of 200 μg/mL. Working standard solutions were prepared by further dilution with methanol. All standard solutions were stored at 4 °C until used. Data were processed using Origin software (OriginLab, Northampton, MA, USA).

### 3.2. Optimization of High-Throughput Field Matrix-Induced Sugaring-Out

A 4 × 2 × 4 × 3 array (sample amount × ratio of ACN:H_2_O × standing time × replicates) was used for the optimization of parameters. Honey samples (0.2, 0.3, 0.4, and 0.5 g) were weighed in 96-deepwell plate and fortified with standards (1.6 μg for HMF, 12 μg for protocatechuic acid, 4 μg for pinocembrin, caffeic acid, and 3,4-dimethoxycinnamic acid, and 8 μg for other phenolic compounds and IS). Then, different volumes of H_2_O (300, 400, 500, and 600 μL) were added. After mixing for 2 min by aspirating in-out using multichannel pipette, different volumes of ACN (700, 600, 500, and 400 μL) were added and mixed again for 1 min. After standing for different times (5, 15, 25, and 35 min) for phase separation, the extractive solution in the upper phase was collected for HPLC analysis.

### 3.3. Optimal Protocol of High-Throughput Field Matrix-Induced Sugaring-Out

Honey samples (0.3 g) were weighted in 96-deepwell plate, and 10 μL of IS (1 mg/mL) was added. Then, 300 μL of H_2_O was introduced. After mixing for 2 min by aspirating in-out using multichannel pipette, 700 μL of ACN was added and mixed for 1 min. Then, the mixture stood with 25 min for phase separation. Finally, the extractive solution in the upper phase was collected for HPLC analysis.

### 3.4. HPLC Analysis

HPLC analysis was performed on Shimadzu (Kyoto, Japan) LC-20AT pump, with SIL-20AC autosampler, CTO-20AC column oven, and SPD-M20A photodiode array detector. An Intersil ODS-3 (GL Sciences, Kyoto, Japan) C18 column (5 μm, 4.6 mm × 250 mm) was used for the separation. The mobile phase was water with 0.1% (*v*/*v*) formic acid (solvent A) and ACN with 0.1% (*v*/*v*) formic acid (solvent B). The gradient elution was as follows: 10% B at 0–5 min, 10% to 26% B at 5–10 min, 26% to 50% B at 10–57 min, 50% to 100% B at 57–58 min, 100% to 10% at 58–60 min, and stayed at 10% for 10 min. The flow rate was 1 mL/min, the injection volume was 5 μL, the column temperature was 30 °C, and the detection wavelength was set at 280 nm.

### 3.5. Method Validation

Validation was carried out according to ICH guideline [38]. Quantifications of analytes were based on calibrations of internal standards. Five-level calibration curves were constructed by the ratio of peak area (analyte/IS) versus the ratio of weight (analyte/IS). The y-intercept was set to 0, and linear fitting was performed to evaluate the linearity. Limits of detection (LODs) and limits of quantification (LOQs) were estimated in spiked honey sample. Accuracy and precision were investigated in the recovery experiment with three fortification levels using 3 × 6 array (levels × replicates) in 96-deepwell plate. The relative standard deviation (RSD) in repeatability (intra-day precision, n = 6) and intermediate precision (inter-day precision, three consecutive days, n = 18) were calculated.

### 3.6. Real Samples Analysis

For commercial honey samples, a 12 × 3 array (samples × replicates) in 96-deepwell plate was prepared as described in Section 3.3. Samples were weighed using a portable balance and prepared outdoors. For in-field honey samples, six colonies in the apiary were investigated. About 2 g of honey were directly collected from beehives using a syringe for each colony. Then samples were weighed using a portable balance and prepared in-field using a 6 × 3 array (samples × replicates) in 96-deepwell plate as described in Section 3.3.

## 4. Conclusions

In summary, a simple and fast high-throughput field MISO method was developed for the simultaneous determination of multiple analytes in honey, including the HMF and nine phenolic compounds. The optimization of the method parameters was systematically and rapidly investigated by constructing sample array in 96-deepwell plate. The proposed method was successfully applied for the outdoor preparation of commercial honey samples and the field preparation of raw honey samples in apiary. The present study provides a simple, rapid, and instrument-free field preparation method for honey sample. This proposed method would be valuable for the quality control of honey and for the design of field and on-site sample preparation.

## Figures and Tables

**Table 1:** Linearity, LOD, and LOQ of the ten analytes.

Analytes	Linear Equation	Linearity Range (μg/mL)	r^2^	LOD (μg/g)	LOQ (μg/g)
5-Hydroxymethylfurfural	y = 2.1198x	1.44–40	0.9998	0.18	0.54
Protocatechuic acid	y = 0.3618x	0.76–21	0.9997	0.90	4.04
Pinocembrin	y = 0.9226x	0.09–2.5	0.9997	0.17	0.51
Caffeic acid	y = 0.8331x	0.18–5	0.9996	0.51	1.02
3,4-Dimethoxycinnamic acid	y = 0.7344x	0.22–6	0.9994	0.39	1.17
Quercetin	y = 0.2954x	0.72–20	0.9992	0.69	4.14
Apigenin	y = 0.6312x	0.54–15	0.9996	0.83	2.50
Kaempferol	y = 0.3666x	0.72–20	0.9989	1.35	4.04
Chrysin	y = 0.9792x	0.36–10	0.9997	0.35	1.57
Galangin	y = 0.4508x	0.58–16	0.9993	1.01	3.03

**Table 2:** Accuracy and precision in spiked honey sample at three spiked levels.

Analytes	Spiked Levels (μg/g)	Intra-Day						Inter-Day	
		Day 1		Day 2		Day 3			
		Recovery (%) ± SD (n = 6)	RSD (%)	Recovery (%) ± SD (n = 6)	RSD (%)	Recovery (%) ± SD (n = 6)	RSD (%)	Recovery (%) ± SD (n = 6)	RSD (%)
5-Hydroxymethylfurfural	5.7	98.63 ± 2.40	2.44	101.99 ± 3.99	3.91	97.85 ± 2.06	2.11	99.49 ± 3.32	3.34
	11.4	104.96 ± 4.14	3.94	107.97 ± 3.63	3.36	102.07 ± 4.25	4.16	105.00 ± 4.51	4.30
	28.6	102.07 ± 3.28	3.22	96.33 ± 1.67	1.73	97.93 ± 3.50	3.57	98.78 ± 3.71	3.76
Protocatechuic acid	4.2	88.13 ± 1.75	1.98	88.83 ± 1.70	1.91	89.78 ± 1.58	1.77	88.91 ± 1.72	1.94
	8.3	83.98 ± 1.68	2.00	89.06 ± 1.97	2.21	85.86 ± 2.42	2.82	86.30 ± 2.89	3.35
	20.8	86.98 ± 1.72	1.98	85.22 ± 3.41	4.00	84.14 ± 3.49	4.15	85.45 ± 3.05	3.57
Pinocembrin	0.5	92.58 ± 2.19	2.37	95.22 ± 1.71	1.79	96.56 ± 1.53	1.59	94.79 ± 2.42	2.55
	1.0	94.74 ± 2.00	2.12	97.04 ± 2.92	3.00	95.20 ± 3.37	3.54	95.66 ± 2.84	2.97
	2.4	100.45 ± 1.90	1.89	96.54 ± 3.38	3.50	97.67 ± 2.66	2.72	98.22 ± 3.06	3.11
Caffeic acid	1.1	95.61 ± 2.80	2.92	96.02 ± 2.65	2.76	96.16 ± 2.80	2.92	95.93 ± 2.59	2.70
	2.2	95.77 ± 2.73	2.85	96.40 ± 3.76	3.90	95.58 ± 2.76	2.89	95.92 ± 2.95	3.08
	5.5	92.87 ± 1.29	1.39	92.78 ± 3.65	3.93	93.37 ± 3.20	3.43	93.00 ± 2.74	2.94
3,4-Dimethoxycinnamic acid	1.2	105.17 ± 3.84	3.65	106.44 ± 2.96	2.78	105.75 ± 1.82	1.72	105.79 ± 2.86	2.70
	2.3	105.43 ± 3.09	2.93	110.38 ± 4.06	3.68	106.99 ± 2.99	2.79	107.60 ± 3.85	3.58
	5.8	106.51 ± 0.96	0.90	102.60 ± 2.23	2.17	103.66 ± 2.14	2.07	104.25 ± 2.44	2.34
Quercetin	4.0	113.50 ± 4.51	3.98	117.11 ± 3.66	3.13	111.77 ± 3.44	3.08	114.13 ± 4.32	3.79
	8.0	104.75 ± 3.64	3.48	100.29 ± 4.18	4.17	107.06 ± 1.18	1.10	104.03 ± 4.22	4.06
	20.2	96.07 ± 3.69	3.84	93.60 ± 3.34	3.57	98.28 ± 3.39	3.45	95.99 ± 3.81	3.97
Apigenin	3.1	102.87 ± 3.92	3.81	102.36 ± 1.88	1.84	96.35 ± 2.60	2.69	100.53 ± 4.10	4.08
	6.1	101.77 ± 4.86	4.78	95.01 ± 1.54	1.62	94.19 ± 3.23	3.43	96.99 ± 4.79	4.94
	15.4	99.52 ± 4.37	4.39	95.21 ± 2.32	2.44	94.71 ± 3.03	3.20	96.48 ± 3.85	4.00
Kaempferol	3.8	103.70 ± 3.45	3.33	101.69 ± 3.67	3.61	99.74 ± 3.86	3.87	101.71 ± 3.82	3.76
	7.7	103.59 ± 3.10	2.99	99.96 ± 2.65	2.65	100.95 ± 3.15	3.12	101.50 ± 3.21	3.16
	19.2	102.36 ± 3.96	3.87	98.02 ± 2.06	2.10	100.65 ± 3.02	3.00	100.34 ± 3.45	3.44
Chrysin	2.0	105.11 ± 2.98	2.84	101.20 ± 3.18	3.14	100.36 ± 2.99	2.98	102.23 ± 3.57	3.49
	4.0	104.70 ± 3.18	3.03	105.44 ± 2.32	2.32	104.57 ± 2.22	2.12	104.91 ± 2.51	2.40
	10.2	101.48 ± 1.99	1.96	106.82 ± 4.42	4.14	103.67 ± 2.30	2.22	103.99 ± 3.68	3.54
Galangin	2.9	104.95 ± 2.48	2.36	107.77 ± 2.75	2.55	104.59 ± 2.61	2.50	105.77 ± 2.86	2.71
	5.9	106.67 ± 2.84	2.66	108.00 ± 2.47	2.28	103.81 ± 3.49	3.36	106.16 ± 3.32	3.12
	14.7	109.74 ± 2.72	2.48	104.54 ± 1.63	1.56	105.68 ± 2.91	2.76	106.65 ± 3.28	3.07

**Table 3:** Results of detected compounds in the real honey samples.

Sample ID	Detected Compounds (μg/g, Mean ± SD, n = 3)				
	5-Hydroxymethylfurfural	Protocatechuic Acid	Caffeic Acid	3,4-Dimethoxycinnamic Acid	Kaempferol
C1	34.96 ± 3.25	-	-	-	-
C2	9.67 ± 1.11	-	-	-	-
C3	6.14 ± 0.17	-	-	-	-
C4	20.14 ± 0.15	-	-	-	-
C5	8.21 ± 0.47	-	-	-	-
C6	85.88 ± 1.07	-	1.30 ± 0.02		-
C7	43.23 ± 1.65	<LOQ	-	-	-
C8	14.16 ± 1.04	-	-	-	-
C9	1.85 ± 0.12	-	-	-	-
C10	72.07 ± 0.54	-	-	-	-
C11	18.16 ± 1.35	-	-	-	-
C12	27.97 ± 0.54	-	-	-	<LOQ
R1	<LOQ	-	-	-	-
R2	<LOQ	-	-	<LOQ	-
R3	<LOQ	-	-	<LOQ	-
R4	<LOQ	-	-	2.02 ± 0.03	-
R5	<LOQ	-	-	<LOQ	-
R6	<LOQ	-	-	-	-

C1–C12: commercial honey samples; R1-R6: raw honey samples; LOQ: limit of quantification; -: not detected.

## Data Availability

Data are contained within the article.

## Sample Availability

Not available.
